# PAX1 methylation as a robust predictor: developing and validating a nomogram for assessing endocervical curettage (ECC) necessity in human papillomavirus16/18-positive women undergoing colposcopy

**DOI:** 10.1186/s13148-024-01691-1

**Published:** 2024-06-07

**Authors:** Yingnan Lu, Haiyue Wu, Kun Fu, YuFei Shen, Lucia Li, Zexi Liao, Yingzhen Liu, Yanan Kang, Yu Zhang

**Affiliations:** 1 Department of Gynecology, Xiangya Hospital, Central South University, 87 Xiangya Road, Changsha, 410008 Hunan China; 2 Gynecological Oncology Research and Engineering Center of Hunan Province, 87 Xiangya Road, Changsha, 410008 Hunan China; 3 National Clinical Research Center for Geriatric Disorders, Xiangya Hospital, Central South University, 87 Xiangya Road, Changsha, 410008 Hunan China

**Keywords:** Endocervical curettage, Human papillomavirus, DNA methylation, PAX1, Nomogram prediction model

## Abstract

**Objective:**

The major challenge in routine endocervical curettage (ECC) among Human Papillomavirus (HPV) 16/18-positive patients is that only a small fraction benefit. Nevertheless, current reported models often overestimate the validity and necessity of ECC, making it difficult to improve benefits for patients. This research hypothesized that assessing paired boxed gene 1 methylation levels (PAX1^m^) and clinical characteristics could enhance the predictive accuracy of detecting additional high-grade squamous intraepithelial lesions or worse (HSIL +) through ECC that were not identified by colposcopy-directed biopsy (CDB).

**Methods:**

Data from 134 women with HPV16/18 positivity undergoing CDB and ECC between April 2018 and April 2022 were collected and analyzed. Quantitative methylation-specific polymerase chain reaction (qMSP) was utilized to measure PAX1^m^, expressed as ΔCp. Univariate and multivariate regression analyses were conducted to screen variables and select predictive factors. A nomogram was constructed using multivariate logistic regression to predict additional HSIL + detected by ECC. The discrimination, calibration, and clinical utility of the nomogram were evaluated using receiver operating characteristic curves (ROC) and the calibration plot.

**Results:**

Age (odds ratio [OR], 5.654; 95% confidence interval [CI], 1.131–37.700), cytology (OR, 24.978; 95% CI, 3.085–540.236), and PAX1 methylation levels by grade (PAX1^m^ grade) (OR, 7.801; 95% CI, 1.548–44.828) were independent predictive factors for additional detection of HSIL + by ECC. In HPV16/18-positive women, the likelihood of additional detection of HSIL + through ECC increased with the severity of cytological abnormalities, peaking at 43.8% for high-grade cytological lesions. Moreover, when cytological findings indicated low-grade lesions, PAX1 methylation levels were positively correlated with the additional detection of HSIL + by ECC (*P* value < 0.001). A nomogram prediction model was developed (area under curve (AUC) = 0.946; 95% CI, 0.901–0.991), demonstrating high sensitivity (90.9%) and specificity (90.5%) at the optimal cutoff point of 107. Calibration analysis confirmed the model’s strong agreement between predicted and observed probabilities.

**Conclusion:**

The clinical nomogram presented promising predictive performance for the additional detection of HSIL + through ECC among women with HPV16/18 infection. PAX1 methylation level could serve as a valuable tool in guiding individualized clinical decisions regarding ECC for patients with HPV 16/18 infection, particularly in cases of low-grade cytological findings.

**Supplementary Information:**

The online version contains supplementary material available at 10.1186/s13148-024-01691-1.

## Introduction

The occurrence of cervical cancer and its precursor lesions, especially high-grade squamous intraepithelial lesions/cervical intraepithelial neoplasia grades 2 to 3 (HSIL/CIN2-3), is strongly associated with high-risk human papillomavirus (hrHPV) infections, of which HPV16 and 18 are the most common oncogenic types [1]. A study with a 10-year follow-up showed that a cumulative risk of progression to CIN3 of 20.7% for women infected with HPV 16, 17.7% for women with HPV 18 infection, and only 3% for women with other hrHPV genotypes [2]. Refining the screening management process is critical to ensuring that high-risk individuals could receive appropriate care. Currently, the medical institutions in China follow the internationally recognized three-step screening method: women with abnormal cervical thin-layer liquid-based cytology and/or hrHPV-positive screened out by primary screening are referred directly for colposcopy, which is known as the second step. Colposcopy-directing biopsy (CDB) and endocervical curettage (ECC), as the third step, can be performed at the colposcopic stage to provide histopathological diagnosis for cervical lesions [3]. If the lesion tissue within the cervical canal cannot be excluded, ECC provides an access for obtaining pathologic tissue to diagnose occult high-grade squamous intraepithelial lesions or more severe ones (HSIL +) that cannot be adequately detected by colposcopy.

However, the effectiveness of ECC in the assessment of cervical lesions and the indications for performing ECC have remained controversial, and routine ECC is evidently impractical because of the controversy and the consequent additional cost [4, 5]. Some research supported that ECC could significantly increase the effectiveness of diagnosing HSIL + . It has been reported that performing only CDB under colposcopy resulted in a 30%–50% missed diagnosis rate for HSIL + [6, 7], while the combination of CDB and ECC could increase the detection rate of cervical precancerous lesions or cervical cancer by 2–19% [8–10]. Liu et al. also discovered that the diagnosis rate of HSIL by ECC was notably elevated among women with HPV 16/18 and high-grade cytology [11]. Therefore, colposcopists are more inclined to perform ECC, especially for patients with HPV16/18 positivity. Nevertheless, although ECC could increase the detection rate of HSIL + , its specificity was not high. In reality, routine ECC only benefited 7–35% of women with HPV16/18 infection [12–14]. A study reported that even when HPV16/18 was positive, the probability of cervical lesions with CIN2 + was only 26.7% if cytology was negative or low-grade [15]. Obviously, for the others with negative cytological results, ECC could be omitted, as treatment is not recommended clinically for lesions less severe than CIN2. Excessive ECC exposed patients to additional costs and risks of postoperative damage, including pain, cervical stenosis, adhesions, and endometriosis, and reduced the probability of attending follow-up appointments [16–18]. As with other invasive procedures, ECC carried inherent risks, including the potential for infection and bleeding. Accordingly, the need for routine ECC should be carefully discussed in the HPV16/18-positive population.

At present, a consensus has been formed that the referred populations for ECC should be highly selective. Previous studies demonstrated that the risk of detecting HSIL + by ECC was age-related, with the sensitivity of ECC increasing for women aged 40 years and older [19, 20]. It was reported that women aged 30 years or older with cytology of atypical squamous cells of undetermined significance (ASC-US) or low-grade squamous intraepithelial lesions (LSIL) and unsatisfactory colposcopy were also recommended to undergo ECC routinely [11]. And indicators such as colposcopic impressions could improve the predictive accuracy for positive results of ECC [21]. Meanwhile, although several predictor-based ECC prediction models were reported in previous studies [20, 21], the performance of these models was not satisfactory as they only took into account the grade of cervical intraepithelial neoplasia identified by ECC, neglecting comparisons with CDB lesion results. In other words, these studies did not point out ECC’s capability in additional detection of CIN. For instance, even if the ECC indicated HSIL, the procedure could be bypassed if the CDB results indicated a more severe condition. To date, there were no studies reporting good models for predicting the probability of additionally detecting HSIL + by ECC, which was more clinically relevant.

In addition, the triage tests (such as p16/Ki67 dual staining or methylation markers) were proposed to reduce unnecessary ECC and refine the patient management process by the American Society for Colposcopy and Cervical Pathology in their 2023 guidelines [16]. A meta-analysis including seven studies and 1055 patients found that PAX1 methylation was associated with the transition of normal tissue to CIN and cervical cancer [22], and the basis of our previous study confirmed its ability to act as a biomarker to triage CIN3 + patients [23]. Furthermore, PAX1 methylation test samples can be collected before colposcopy, providing available results to be considered when making decisions that whether ECC is necessary. In this study, we hypothesized that PAX1 methylation could play a crucial role in predicting ECC necessity. We developed and validated a model to predict additional detection of HSIL + through ECC by using PAX1 methylation level, cytology and age, aiming at promoting precision medicine and individualized screening for patients referred for colposcopy due to already available detection of HPV16/18 positivity.

## Methods

### Study design, setting and participants

This was a cross-sectional study in which we reviewed anonymized data from all patients who were referred for colposcopy and underwent both CDB and ECC at the Department of Gynaecology, Xiangya Hospital, Central South University, due to HPV16/18-positive with or without abnormal cytology, between April 2018 and April 2022. The study adhered to the TRIPOD statement for reporting [24]. The data collected included demographic information, HPV status, cytological results, PAX1 methylation level, colposcopic impressions, and pathological findings from CDB and ECC. Demographic data such as age, menopause, and medical history were obtained from electronic medical records for all study subjects. Exclusion criteria were (1) the time of PAX1 methylation test was too far from the time of biopsy (more than 2 weeks), (2) invalid PAX1 methylation result, (3) history of cervical physiotherapy (laser or photodynamic therapy), (4) history of surgical procedures (circumferential electrodesection, cold-knife conization, or hysterectomy), or history of pelvic radiotherapy, (5) absence of diagnosis, and (6) incomplete charts. The study was approved by the Ethics Committee of Xiangya Hospital, Central South University (number 2018121117).

Exfoliated cervical cells were collected and stored in ThinPrep vials (Hologic, USA) for cytological tests (TCT) conducted by two cytopathologists. Cytological results were reported according to the revised Bethesda nomenclature [25], with negative intraepithelial lesion or malignancy (NILM) defined as a negative cytological result, and atypical squamous cells of undetermined significance [ASC-US], low-grade squamous intraepithelial lesions [LSIL], atypical squamous cells cannot exclude high-grade squamous cells of undetermined signification [ASC-H], and high-grade squamous intraepithelial lesions [HSIL], and squamous cell carcinoma [SCC] were considered to be abnormal cytological results. Due to the small sample size in the subgroups, in statistical analysis, ASC-US and LSIL were categorized as ≤ LSIL, while ASC-H, HSIL and SCC were categorized as > LSIL. Cobas 4800 test (Roche, USA), HPV Polymerase Chain Reaction (HPV PCR; Genetel Pharmaceuticals, China) were used as tests for HPV. Cobas 4800 tested for HPV16 and HPV18, as well as 12 other high-risk types (HPV31, 33, 35, 39, 45, 51, 52, 56, 58, 59, 66, 68). The HPV PCR test included 13 hrHPV genotypes, including HPV16, 18, 31, 33, 35, 39, 45, 51, 52, 56, 58, 59, which were independently developed by China and approved by the State Food and Drug Administration (SFDA). The subjects included in this study had HPV test results of HPV16( +) and/or HPV18( +) regardless of other HPV types, the HPV type and test method were not recorded for each patient. All cytology and HPV tests were performed at the hospital in accordance with the procedures provided by the vendor.

Collected exfoliated cervical cells were preserved in phosphate-buffered saline (PBS) solution at  − 20 °C until assayed. Genomic DNA (gDNA) was extracted using QIAamp DNA Mini Kit (Qiagen, Germany). NanoMicroDroplet 2000 Spectrophotometer (Thermo Fisher Scientific, USA) was used to assess the purity and concentration of gDNA. All methylation assays were performed by (HOOMYA, China) certified company. Methylation-specific quantitative PCR (qMSP) was used to determine the methylation levels of PAX1 (PAX1^m^) using TaqMan-based technology in a Lightcycler LC480 system (Roche Applied Science, Germany) with the VIC gene as an internal reference. The cross point (Cp) values for PAX1 and VIC could be determined in each sample. The Cp value for VIC should be less than 35; otherwise, a retest was necessary. The calculation of DNA methylation status involved the difference between the Cp values of the target gene and the reference gene (ΔCp = CpPAX1 − CpVIC), where lower ΔCp values indicated higher levels of methylation. Positive and negative controls were established using Caski and C33A cancer cell lines.

Colposcopy was performed under electronic colposcopy (3ML, LEISEGANG, China) by an experienced colposcopy specialist following a standard procedure that included a visual assessment of the cervix, including its visibility, transformation zone (TZ) type (I / II / III), acetic acid white changes (none, thin, thick) and colposcopic impressions (normal/benign, low grade, high grade, and cancer) [26, 27]. Colposcopy was considered satisfactory if all the transformation areas of cervical epithelium and the lesions were completely visible under colposcopy; otherwise, it was considered unsatisfactory. Multipoint biopsy was performed at the lesion site for those with suspicious lesions detected during satisfactory colposcopy, and at the squamocolumnar junction at random 4-quadrant punch biopsies were taken for those with unsatisfactory colposcopy. Endocervical tissue was routinely extracted by ECC using a Kevorkian spatula after biopsy, and the specimens were sent for pathological examination.

### Selection of predictors

The model predictors were divided into three main categories: general information, laboratory tests obtained before colposcopy and observations during colposcopy, which were established through an exhaustive literature review and consultation with clinical experts. All risk factors reported to be associated with ECC detection of HSIL + as well as the additional detection of HSIL + from ECC were incorporated, including age, cytology, cervical visibility, TZ type and colposcopic impression. Considering the clinical practicability of the model, variables like menopausal status, cervical atrophy, gravidity, parity, contraceptive method, acetowhite changes, and Lugol staining were also included in the study. Moreover, given the documented high screening accuracy of methylation for cervical high-grade squamous intraepithelial lesions or worse (HSIL +), particularly the effectiveness of paired boxed gene 1 (PAX1) methylation as a biomarker for detecting CIN3 or worse (CIN3 +), we explored the clinical utility of PAX1 methylation in preoperative ECC triage. PAX1 methylation was included as a predictor, and its correlation as a dichotomous variable (hypermethylation, hypomethylation) with the additional detection of HSIL + from ECC was discussed. The coding of these variables is outlined in Supplementary Table 1.

### Outcome definition

All colposcopy, CDB and ECC operations were performed by the same gynecologist. Pathological diagnoses were independently assessed and confirmed by two experienced pathologists blinded to the cytology findings, HPV testing results and colposcopy impression. In cases of disagreement, resolution involved a third senior physician, and challenging cases underwent immunohistochemical staining for p16 and Ki67. The histological diagnosis was graded as NILM, LSIL, HSIL, SCC, AIS or AC according to the Lower Anogenital Squamous Terminology Standardization Project for HPV-Associated Lesions (LAST) and the WHO Classification of Tumors of the Female Reproductive Organs (4th edition) [28, 29]. LSIL indicated cervical intraepithelial neoplasia grade (CIN1), and HSIL included CIN2 and CIN3. The final diagnosis relied on the more severe pathological findings from CDB and ECC. Outcomes were defined as (1) Cases with pathological findings detected by ECC were less severe than or equal to CDB [ECC ≤ CDB], (2) Cases with the pathological findings detected by ECC were more severe than CDB, with ECC detecting HSIL and worse [ECC (H +) > CDB], and (3) Cases in which pathological findings identified by ECC were more severe than CDB, with ECC detecting LSIL [ECC (L) > CDB]. The second type of patient is used as a round truth for training and validation of machine learning models, involved in the development of clinical predictive models.

### Sample size

Currently, there was no standard method for calculation of sample size. In this study, the sample size depended on availability of data. With 13 candidate predictors, there were nine events for each variable.

### Statistical analysis

In summary statistics, as all the continuous variables were nonnormal, and medians and interquartile ranges (IQR) were employed to describe data distribution. Frequency with percentage was used to describe data distribution of categorical variables. Mann–Whitney U test and χ2 test were used to explore the difference of clinicopathological characteristics between groups. Cutoff points of PAX1^m^ grade and age were determined by selecting the points with the highest Youden indexes on the ROC curves, when ΔCp values of PAX1 methylation and the value of age were involved in univariate analyses. Based on the hypothesis of missing at random, incomplete observations were imputed with multiple imputation, and one imputed data set was established with SPSS 27.0. Univariate analyses were applied to all mentioned variables, and the criteria for variables being included in multivariate analyses was *P* < 0.05 in univariate analyses. Corresponding 95% confidence interval (95% CI) and odds ratio (OR) were used to demonstrate the correlations between the predictors and outcome. For development of the prediction models, R 4.2.3 was used for univariate and multivariate logistic regression analyses, establishment of receiver operating characteristic (ROC) curves and nomograms, as well as calculation of AUC values. Bootstrap method (*n* = 1000) was used to validate the prediction models, and calibration plots were utilized as visualized tools to describe the coincidence between predicted value and observed results [30]. Statistically significance referred to two-sided *P* < 0.05 in all statistical analyses.

## Results

### Patient demographic and clinical characteristics

**Fig. 1:**
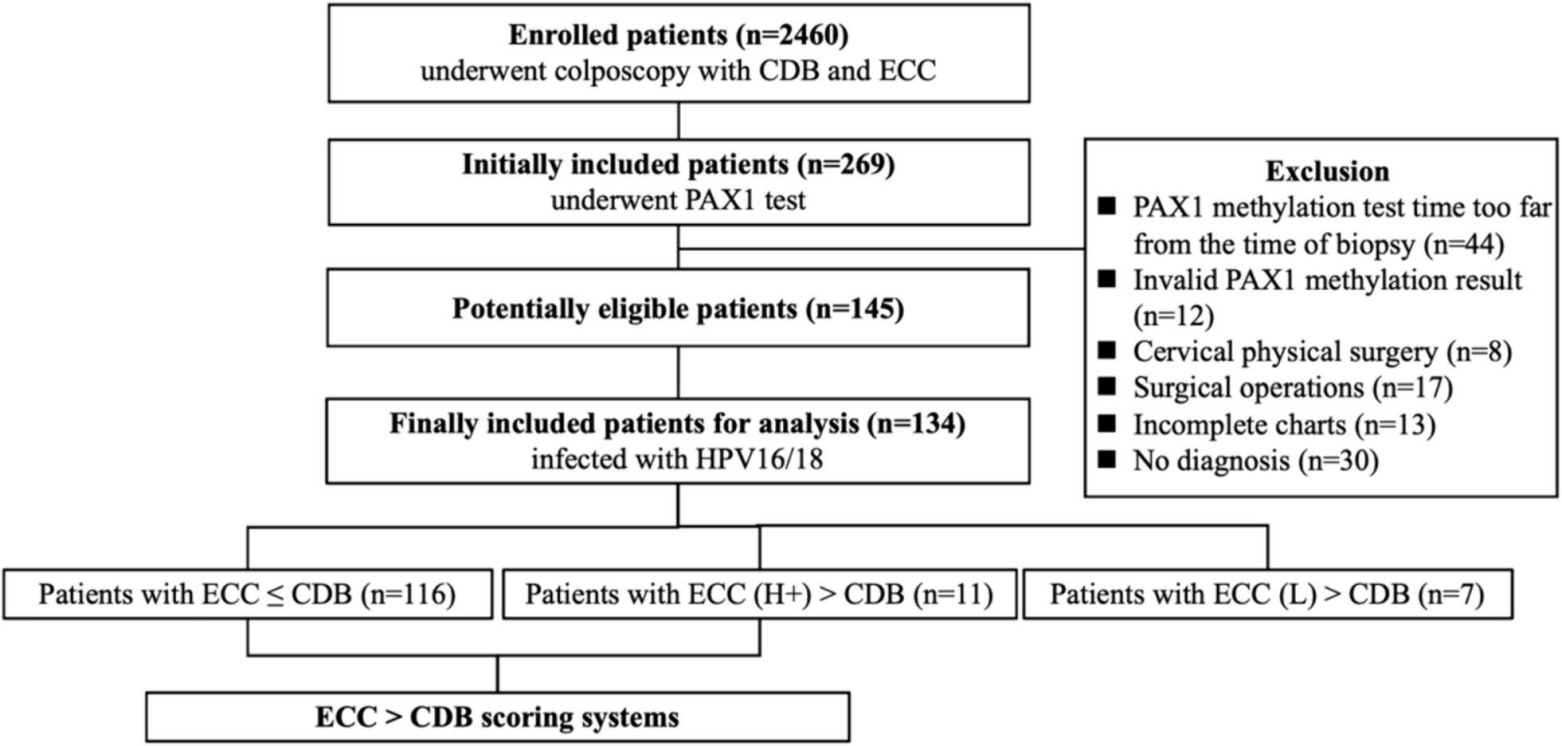
Flowchart of patients. CDB, colposcopically directed biopsy; ECC, endocervical curettage; PAX1, paired boxed gene 1; HPV16/18, human papillomavirus 16/18; LSIL, low-grade squamous intraepithelial lesion; HSIL + , high-grade squamous intraepithelial lesion or worse; ECC ≤ CDB, cases with pathological findings detected by ECC were less severe than or equal to CDB; ECC (H +) > CDB, cases with the pathological findings detected by ECC were more severe than CDB, with ECC detecting HSIL and worse; ECC (L) > CDB, cases in which pathological findings identified by ECC were more severe than CDB, with ECC detecting LSIL, PAX1 methylation test time apart from biopsy time greater than 2 weeks was defined as too far

A total of 2460 patients who completed both CDB and ECC due to cervical HPV16/18 infection from April 2018 to April 2022 were included; initially, 269 of them completed PAX1 methylation testing before biopsy. According to our demonstrated exclusion criteria, 124 patients were excluded. Finally, 134 patients who underwent CDB and ECC due to HPV16/18 infection patients with or without abnormal TCT results were included into statistical analyses. Among their test results, 116 (86.57%) showed ECC ≤ CDB, and 18 (13.43%) displayed ECC > CDB. In the ECC > CDB population, 11 patients had ECC pathological results of HSIL + , while the pathologic results of ECC were LSIL in the other 7 cases (Fig. 1). Among patients with ECC (H +) > CDB, one patient (9.1%) had NILM, three patients (27.3%) had lesions ≤ LSIL, and seven patients (63.6%) had lesions > LSIL. It was worth mentioning that we focused on necessity of performing ECC in this study. Patients with LSIL lesions would not be recommended for any clinical intervention; therefore, they were not the target population for ECC. Consequently, the analysis of prediction model did not include this group of seven patients. However, since they might be a population who needed closely monitoring, their clinical characteristics were described in followed discussion. Table 1 compared the distribution of demographic and clinical characteristics of ECC ≤ CDB with ECC (H +) > CDB groups. The median age was 40 (IQR 30–50) years among the ECC ≤ CDB group, and 53 (IQR 48.5–57.5) years among the ECC (H +) > CDB group.

**Table 1:** Clinicopathologic characteristics of ECC ≤ CDB and ECC (H +) > CDB patients/demographics and clinicopathologic characteristics of study population (*N* = 127)

Characteristics	ECC ≤ CDB^a^(n = 116)	ECC(H +) > CDB^b^(n = 11)	${\varvec{\chi}}$^2^	*P*-valve
Age^c^ (year)	40 (20)	53 (9)		0.008
Age group (year)			9.011	0.003
≤ 50	83 (65.4%)	3 (2.4%)		
> 50	33 (26.0%)	8 (6.3%)		
Menopause			2.965	0.085
No	82 (64.6%)	5 (3.9%)		
Yes	34 (26.8%)	6 (4.7%)		
Contraceptive method			0.637	0.959
None	75 (59.1%)	7 (5.5%)		
Condom	17 (13.4%)	1 (0.8%)		
Contraceptive rings	9 (7.1%)	1 (0.8%)		
Ligation	14 (11.0%)	2 (1.6%)		
Drug	1 (0.8%)	0 (0)		
Gravidity^a^	3 (2)	3 (3)		0.316
Parity	1 (1)	2 (1)		0.087
TCT			29.623	< 0.001
NILM	69 (54.3%)	1 (0.8%)		
≤ LSIL	38 (29.9%)	3 (2.4%)		
> LSIL	9 (7.1%)	7 (5.5%)		
PAX1^m^ group			26.158	< 0.001
< 6	11 (9.1%)	7 (5.8%)		
≥ 6	100 (82.6%)	3 (2.5%)		
Cervix visibility			0.193	0.661
Inadequate	2 (1.6%)	0 (0)		
Adequate	114 (89.8%)	11 (8.7%)		
Cervical atrophy			0.193	0.661
No	114 (89.8%)	11 (8.7%)		
Yes	2 (1.6%)	0 (0)		
TZ type			1.394	0.498
Type I	12 (9.5%)	0 (0)		
Type II	13 (10.3%)	1 (0.8%)		
Type III	90 (71.4%)	10 (7.9%)		
Acetowhite changes			0.423	0.809
None	54 (42.5%)	4 (3.1%)		
Thin	36 (28.3%)	4 (3.1%)		
Thick	26 (20.5%)	3 (2.4%)		
Lugol staining			0.032	0.857
Nonstained	45 (35.7%)	4 (3.1%)		
Stained	70 (55.6%)	7 (5.6%)		
Colposcopic impression			6.875	0.076
Normal/benign	61 (50.4%)	1 (0.8%)		
Low-grade	29 (24.0%)	5 (4.1%)		
High-grade	20 (16.5%)	3 (2.5%)		
Cancer	2 (1.7)	0 (0)		

^a^ECC diagnosis less severe than or equal to CDB

^b^ECC diagnosis more severe than CDB with ECC detecting HSIL and worse (HSIL +)

^c^Statistics presented: Median (IQR); n (%)

### Univariate and multivariate predictors of ECC > CDB and ECC HSIL + 

**Fig. 2:**
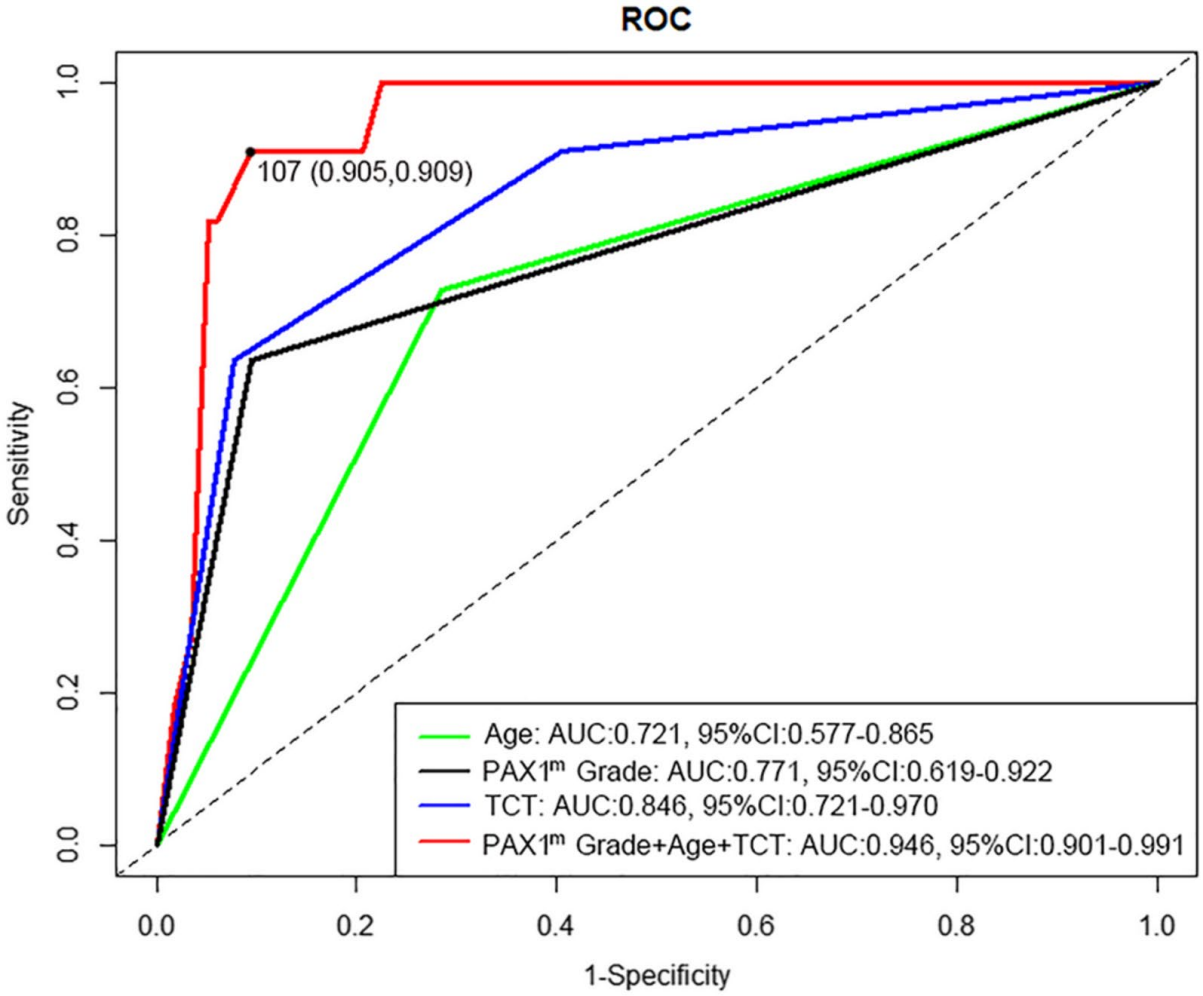
ROC curves of different models predicting ECC (H +) > CDB for women with positive HPV16/18. ROC: receiver operating characteristic; AUC: area under curve; CI: confidence interval; PAX1^m^ Grade: PAX1 methylation level by grade

In univariate analyses, statistical significance was found on PAX1 methylation levels between ECC ≤ CDB group and ECC (H +) > CDB group, as categorical (*P* < 0.001) variables. The increased level of PAX1 methylation indicated a greater opportunity to be additionally detected as HSIL by ECC, and the univariate model based on PAX1^m^ grade showed good performance, with an area under curve (AUC) of 0.770 (Fig. 2). Other statistically significant variables identified by univariate analysis, including age (*P* = 0.003) and TCT (*P* < 0.001), were involved in multivariate logistic analysis for development of clinical prediction scoring system.

In the multivariate logistic regression model with age, PAX1^m^ grade and TCT results, PAX1^m^ grade and TCT remained significant predictors of ECC(H +) > CDB. Compared with patients whose PAX1 methylation levels were low (ΔCp > 6), patients with PAX1 hypermethylation (ΔCp ≤ 6) had a 7.801-fold higher risk (95% CI 1.548–44.828) to have a test result of ECC (H +) > CDB (*P* = 0.0144). The possibility of ECC (H +) > CDB was 5.654 (95% CI 1.131–37.700) times higher in patients older than 50 comparing to those under 50 (*P* = 0.0455). Patients with cytology > LSIL showed significantly higher risk of ECC (H +) > CDB, with OR as 24.978 (*P* = 0.0076). (Table 2).

**Table 2:** Multivariable logistic regression analysis of factors associated with ECC (H +) > CDB

Characteristics	OR (95%CI)	*P*-valve
Age group (year)		
< 50	Reference	
≥ 50	5.654 (1.131,37.700)	0.0455
TCT		
NILM	Reference	
≤ LSIL	3.027 (0.302,67.144)	0.3738
> LSIL	24.978 (3.085,540.236)	0.0076
PAX1^m^ group		
≥ 6	Reference	
< 6	7.801 (1.548,44.828)	0.0171

Then, we further verified the positive correlation between PAX1 methylation level and the additional detection of HSIL + from ECC in patients with TCT results of ≤ LSIL and > LSIL, exploring the role of PAX1 methylation in determining ECC necessity among patients with different outcomes of TCT. We found that PAX1^m^ grade was correlated with ECC (H +) > CDB in patients with TCT as ≤ LSIL, showing a positive correlation (*P* < 0.001) (Table 3). However, no similar results were observed in patients with TCT as > LSIL.

**Table 3:** Correlation between PAX1 methylation level and the additional HSIL + detected by ECC in patients with TCT as ≤ LSIL and > LSIL

PAX1^m^ group	TCT: ≤ LSIL (*n* = 106)				TCT: > LSIL (*n* = 15)			
	ECC ≤ CDB	ECC (H +) > CDB	${\varvec{\chi}}$^2^	*P*-valve	ECC ≤ CDB	ECC (H +) > CDB	${\varvec{\chi}}$^2^	*P*-valve
< 6	7	4	35.900	< 0.001	4	3	0.045	0.833
≥ 6	95	0			5	3		
Total	102	4			9	6		

### Model development and validation

**Fig. 3:**
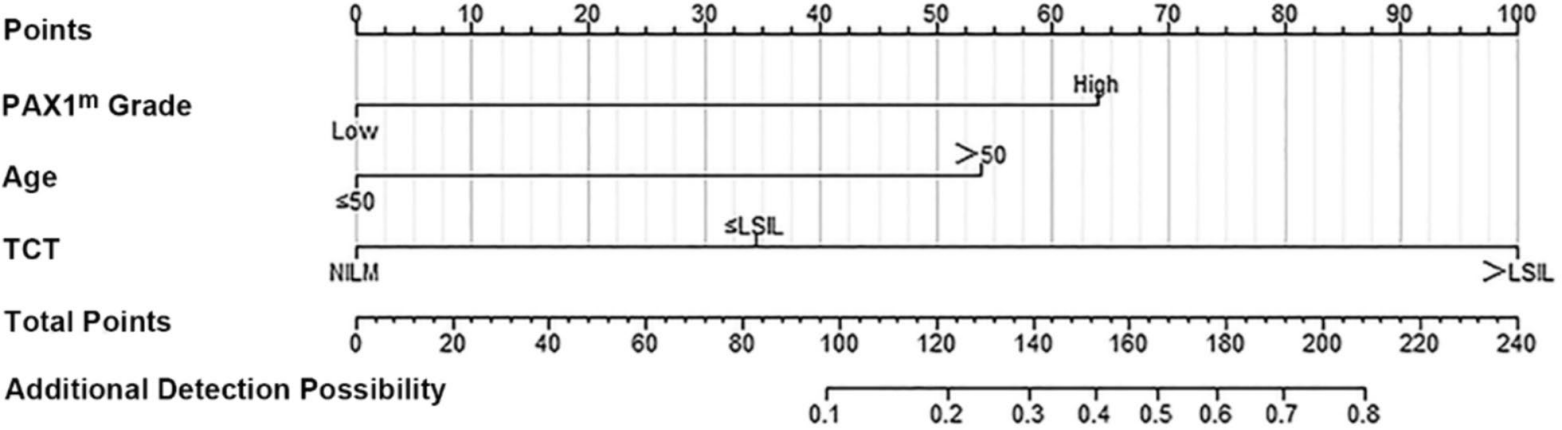
Nomogram predicting ECC (H +) > CDB for women with positive HPV16/18

On the basis of univariate and multivariate analyses, a clinical scoring model with three predictors, including PAX1 methylation grade, age, and TCT was constructed. Figure 2 displays the performance of object classification of our scoring model, and the AUC was 0.946 (95% CI 0.901–0.991). Compared with the univariate model of PAX1 methylation grade, age or cytology that provide lower predictive potential, the multivariate scoring model incorporating clinical characteristics showed obviously better performance (Fig. 2). Figure 3 shows the nomogram of our scoring model, and specific grading value was assigned for every predictor. After determining the grading value of all three predictors, a total score could be calculated to speculate on the possibility of ECC (H +) > CDB for each patient. According to our multivariate ROC curve and nomogram, the cutoff point of our prediction scoring system was 107, which had the highest Youden index.

**Fig. 4:**
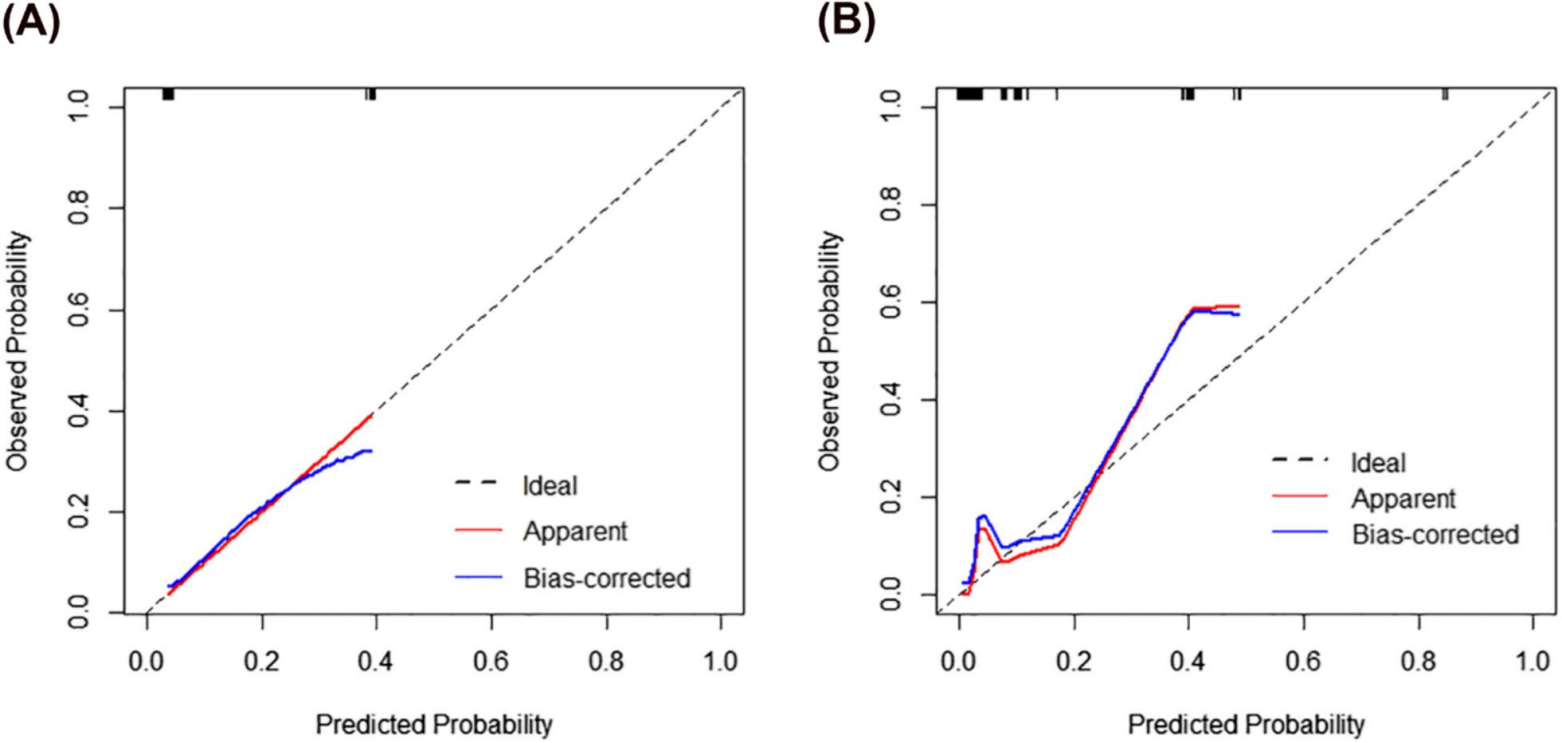
Calibration plots showing the observed frequency and predicted probability for the predictive model (**A**) The univariate mode of PAX1^m^ Grade. (**B**) The multivariate mode

Internal validation was assessed by bootstrapping (n = 1000) and tenfold cross-validation (*n* = 50) [31–33]. The average value of the AUCs was 0.956 (95% CI 0.889–0.982) according to bootstrap procedure, and the modified AUC according to tenfold cross-validation was 0.901. Figure 4 shows the calibration plots of both the univariate model and the multivariate scoring model, displaying the predicted and actual probabilities by bootstrapping. In the univariate model, the predicted possibility was basically accordant with the actual possibility, while the multivariate scoring model showed risk of underestimating the probability of ECC (H +) > CDB.

## Discussion

In recent years, reported ECC prediction models provided clinicians with the basis for decision-making on whether to perform ECC during colposcopy [20, 21]. However, in China, unlike regular outpatient settings, colposcopy clinics were typically run independently and operated by only one physician performing procedures. The substantial workload associated with prediction models utilizing multiple parameters [5–9] made rapid triage for ECC challenging, rendering it less feasible. Our model, in contrast, was highly straightforward, holding greater practical value in clinical use. Additionally, a common limitation of the previously reported models was that they mainly use HSIL + or even LSIL + as the study endpoints to stratify study population, and statistically significant factors were utilized as valid indicators for performing ECC. Nevertheless, a meta-analysis reported that within two years, 47.39% (95% CI 35.92%, 58.86%) of LSIL lesions naturally regressed, only 20.81% (6.08%, 35.55%) of the lesions progressed to HSIL [34]. It was demonstrated in numerous studies that 60% of LSIL lesions naturally regress within one year, 30% persist, and only very few lesions progress to HSIL within two years [27, 35, 36]. Gynecologists consistently managed LSIL cases identified through ECC and cytology (CDB) with an approach involving observation, without immediate treatment [27, 37–39]. Therefore, the conclusion based on ECC for LSIL + as an endpoint might exaggerate the benefits of ECC. In addition, conducting risk factor analysis for ECC-detected HSIL + instead of ECC (H +) > CDB did not clearly indicate whether ECC can be skipped. As these patients encompassed individuals whose CDB results were either worse or similar to ECC, even in cases where ECC results indicated HSIL + , we did not deem additional ECC necessary for such patients.

In this study, our primary focus was on women positive for HPV16/18. In China, HPV16/18 had a higher risk of CIN2 + than other HR-HPV genotypes (30.1% vs. 10.2%, *P* < 0.001). Even among women with low-grade cytology, the risk for HPV16/18 (58.2%) was higher than for other HR-HPVs (16.8%)[15]. When these patients were referred for colposcopy, colposcopists were typically vigilant and often recommended routine ECC to avoid missing occult HSIL + . However, on the one hand, the majority of these patients did not benefit from ECC, on the other hand, they also had to bear the additional burden of its associated trauma and economic costs. Currently, there are no concise, convenient, and economical methods to determine whether ECC can be omitted for these individuals. Therefore, in this study, using ECC > CDB and ECC as HSIL and worse [ECC (H +) > CDB] as the endpoints of interest, we analyzed predictive factors and established a nomogram, aiming to provide an effective for individualizing the assessment of the risk of ECC (H +) > CDB in women positive for HPV16/18. In the real world, colposcopists can estimate the risk value of ECC (H +) > CDB for each patient based on the assigned scores of the aforementioned predictive factors before commencing observation, thereby making informed decisions on whether ECC is necessary.

### Main findings

Among all women who underwent PAX1 methylation testing, we found that setting the PAX1^m^ ΔCp value threshold at 6 had the highest Youden index and the best classification efficacy of the model, ensuring maximum identification of ECC (H +) > CDB (sensitivity of 0.901, specificity of 0.800, and a Youden index of 0.701). The independent predictors of ECC (H +) > CDB were age (*P* = 0.003), TCT (*P* < 0.001), and PAX1 methylation level (*P* < 0.001). The nomogram constructed by incorporating the above three factors was well calibrated and well differentiated, and had the potential to provide a strong basis for decision-making on whether ECC was necessary for the patients referred for colposcopy in clinical practice. The model had an AUC of 0.946 (95% CI 0.901–0.991) for the probability of ECC (H +) > CDB. The ideal cutoff point was 107, sensitivity was 0.905, and specificity was 0.909. Due to the limited scale of data, we performed internal validation, using bootstrapping (*n* = 1000) and tenfold cross-validation (*n* = 50). The tenfold cross-validation reported a modified AUC as 0.901, average value of the AUCs according to bootstrap procedure was 0.956 (95% CI 0.889–0.982).

### Comparison with previous studies

The general consensus is that the age plays a pivotal role as a decision-making indicator for performing ECC. With advancing age, there is a decline in female hormone levels, and the cervix undergoes gradual changes, causing the transformation zone to shift inward to the cervical canal. This alteration poses a challenge for colposcopists in lesion detection. Individuals in such category may gain more benefits from ECC. However, there was still controversy regarding the age threshold. In this study, the research population was classified into two age groups based on the risks of ECC (H +) > CDB. Our findings revealed that patients over the age of 50 had a higher risk value for ECC (H +) > CDB, aligning with the previous clinical report [20]. From a cost–benefit perspective, Shepherd et al. also suggested routine ECC for individuals over 50 [19], whereas mainstream studies questioned the value of routine ECC in younger women, excluding those aged 21–29 might result in the underdiagnosis of approximately 19% of HSIL cases [40]. This suggested that the possibility of additional HSIL + detection through ECC should not be overlooked in younger women, and personalized prediction and management based on other risk factors were necessary.

Cytological results also contributed to prediction of ECC (H +) > CDB. In cases of low-risk lesions (≤ LSIL), the rate of ECC (H +) > CDB was 7.3%, while in high-risk lesions (> LSIL), the rate significantly increased to 43.8%. In the multivariate analysis, cytological findings indicating > LSIL exhibited a higher risk value of ECC (H +) > CDB, on the contrast, lower risk values were observed in patients with ≤ LSIL. In the latter population, deciding whether to proceed with ECC required personalized consideration, taking into account other clinical variables. Our results were similar to a large cross-sectional study of women infected with HPV16/18 [41], and the prevalence of CIN3 + was associated with increased severity of cytologic abnormalities in HPV 16/18-positive women and peaked at cytology HSIL + (89.9% and 82.3%), carrying a significantly higher risk compared to NILM (OR = 65.466, 95% CI 50.234–85.316). However, due to the small sample size, further sub-analysis by cytological results would not be feasible, which limited the precision of the estimates.

We believe that there is necessity for further discussion on predictive factors beyond those previously reported. Incorporating new predictive factors could potentially enhance the model specificity while preserving high sensitivity. For instance, the p16/Ki67 dual staining was approved by the U.S. Food and Drug Administration as an alternative to cytology for cervical cancer screening [5, 42], and ECC would be recommended if the p16/Ki67 dual staining was positive. However, in China, the p16/Ki67 dual staining is currently only applied in the pathological diagnosis of tissues after biopsy. In addition, potential predictive factors also include E6/E7 messenger RNA or E6 oncoprotein. However, due to the cost and the limitation of detection method, it is challenging to expand the application of these predictive factors. Numerous studies highlighted that the methylation, especially PAX1 methylation, had high screening accuracy for CIN2 + , serving as a potential triage method for women with HPV infections or as a predictor of the worst pathological outcome [23, 43–49]. In this study, we also considered the assessment of PAX1 methylation levels. We found that compared to patients with ECC ≤ CDB, patients with ECC (H +) > CDB had smaller PAX1 methylation ΔCp values, indicating higher PAX1 methylation levels. We determined the optimal PAX1 methylation ΔCp cutoff value for referring to ECC as 6, considering ΔCp < 6 as highly methylated. Interestingly, despite the risk of ECC (H +) > CDB was low in patients with TCT as ≤ LSIL, correlation analyses showed that PAX1 methylation discriminated highly between ECC ≤ CDB and ECC (H +) > CDB when the TCT result was low-risk (≤ LSIL) (*P* < 0.001). The univariate model containing only PAX1^m^ could effectively predict ECC(H +) > CDB, and 103 patients (81.1%) could have skipped ECC according to the PAX1^m^ univariate model. In comparison with the univariate model based on PAX1 methylation grading, a multivariate scoring model combining PAX1 methylation with clinical features exhibited significantly better predictive performance (Fig. 2A), and 111 patients (87.4%) could have not undergone ECC if our nomogram was used for clinical triage for HPV16/18-infected women. To our knowledge, this study was the first to evaluate these factors and incorporate them into an ECC prediction model.

In recent years, studies also explored other factors such as cervical canal atrophy, transformation zone type, Lugol staining, acetic acid changes, and colposcopic impressions as potential indicators for the effectiveness of ECC. Unfortunately, our results indicated that these factors were not independent predictors of ECC (H +) > CDB. Previous research suggested that if the transformation zone was partially or entirely not visible, certain lesions, including cancer, may be concealed within the cervical canal, ECC was recommended in this circumstance. However, study conducted across multiple European centers contradicted it by indicating that there was no difference in the ECC detection rates for patients with or without a completely visible squamocolumnar junction (SCJ) [4]. Additionally, research showed that even when the TZ was fully visible under colposcopy, 5% of patients still got positive ECC results [50]. According to the 2023 ASCCP guidelines, the recommendation for ECC when the SCJ is partially or completely invisible relied on the premise that the colposcopic evaluation of the transformation zone was reproducible. If reproducibility was poor, inaccuracies in assessing the transformation zone, which was influenced by the experience and skills of the colposcopist, might lead to the omission or misuse of ECC. In our study, however, the results demonstrated that colposcopic impression was not independent predictor of ECC (H +) > CDB. Therefore, further large-scale studies are needed to determine whether colposcopic assessment results are valuable factors for deciding on ECC.

Our findings provided a basis for the management of colposcopic procedures in HPV16/18-positive patients. It was inconclusive whether CDB only or CDB combining with ECC should be routinely performed in this population. The nomogram presented in this study well differentiated the ECC (H +) > CDB population, recommending ECC for patients with a total score greater than 107, although CDB results may not be serious. Patients with total scores less than 107 might opt for regular follow-up, as the results of ECC might not affect the decision on treatment regimen, which could be determined by the pathological findings of CDB alone. For those with ECC (L) > CDB, we found that after applying this predictive model, their total risk scores were all less than 107 respectively. This implied that their risk of ECC (H +) > CDB was low at this point, and ECC might not be necessary, which aligned with the actual situation. However, it was important to note that one patient in this category had a high level of PAX1 methylation, suggesting that close follow-up with appropriately shortened intervals for her was required.

### Strengths and limitations

Our prediction model’s strengths laid in focusing on approach to improve ECC decision-making process, integrating clinical factors with PAX1 methylation levels—a biomarker not widely adopted in current clinical settings. To the best of our knowledge, few studies have discussed the association between PAX1 methylation and colposcopic features, cytology and ECC results. Therefore, as preliminary data, the results were representative and reliable and can be used to provide additional help in the design of future prospective studies. Additionally, this work marked an important step toward personalized cervical cancer screening strategies, promising to reduce unnecessary procedures while ensuring high-risk individuals receive appropriate care.

Our study had some limitations. First, PAX1 methylation level testing was not a routine item in clinics even though it can be provided by tertiary hospitals in China. Therefore, further research or clinical applications require additional data support. Second, on the one hand, our study was a single-center study, which might restrict the generalizability of the findings. On the other hand, considering data availability, our sample size was relatively small, making it impractical to proportionally divide patients into training and validation sets. As a result, external validation was not conducted, and it may lead to statistical instability. Third, the time span involved was quite extensive, and although the colposcopist performing the procedures was not changed, biases may be introduced due to the long duration. Finally, calibration plots indicated a risk of underestimating the probability of ECC (H +) > CDB in the multivariable scoring model, which may also be related to the small sample size. Therefore, our study results should be considered as preliminary evidence, and future directions could involve external validation of the nomogram in a broader, multi-center cohort to ensure its applicability across diverse populations.

## Conclusion

In summary, a predictive nomogram for additional detection of HSIL + by ECC was established and validated among HPV16/18-positive women, predicting the necessity of ECC in women referred for colposcopy with an acceptable level of discrimination. The nomogram incorporated three demographic and clinical features, and a score greater than 107 should be suggested for ECC, while the ones who got scores less than 107 were not recommended to take ECC routinely. All patients included in our study were recommended to undertake ECC because of HPV16/18 positivity, however, 111 of them (87.4%) could have not undergone ECC if our nomogram was used for clinical triage for HPV16/18-infected women. Especially, when cytology indicated low-grade lesion, PAX1 methylation could serve as an important triage factor and tool to determine whether ECC should be employed. The reliability and practicality of this tool deserves evaluation in prospective studies.

### Supplementary Information

Supplementary Material 1

## Data Availability

The original contributions presented in the study are included in the article/supplementary material. Further inquiries can be directed to the corresponding authors.

## Abbreviations

CDB: Colposcopically directed biopsy
ECC: Endocervical curettage
PAX1: Paired boxed gene 1
PAX1^m^: The methylation levels of PAX1
HPV16/18: Human papillomavirus 16/18
hrHPV: High-risk human papillomavirus
NILM: Negative intraepithelial lesion or malignancy
ASC-US: Atypical squamous cells of undetermined significance
LSIL: Low-grade squamous intraepithelial lesions
ASC-H: Atypical squamous cells cannot exclude high-grade squamous cells of undetermined signification
HSIL: High-grade squamous intraepithelial lesions
SCC: Squamous cell carcinoma
CIN: Cervical intraepithelial neoplasia
TCT: ThinPrep cytologic test
OR: Odds ratio;
ROC: Receiver operating characteristic curves
AUC: Area under curve
CI: Confidence interval
ECC ≤ CDB: Cases with pathological findings detected by ECC were less severe than or equal to CDB;
ECC (H +) > CDB: Cases with the pathological findings detected by ECC were more severe than CDB: with ECC detecting HSIL and worse
ECC (L) > CDB: Cases in which pathological findings identified by ECC were more severe than CDB, with ECC detecting LSIL
