# Supplementary material for: PAX1 methylation as a robust predictor: developing and validating a nomogram for assessing endocervical curettage (ECC) necessity in human papillomavirus16/18-positive women undergoing colposcopy
Source: Clin Epigenetics. 2024 Jun 7;16:77. doi: 10.1186/s13148-024-01691-1 (PMC11157736; doi:10.1186/s13148-024-01691-1)
Supplement: Supplementary file 1 — Supplementary Material 1 [file 13148_2024_1691_MOESM1_ESM.docx]

**Supplementary Table 1** The coding of variables.

| Variable | Categorical/Binary/Continuous | Coding |
| --- | --- | --- |
| Age groups | Categorical | 1=≤50  2=>50 |
| Menopause (yes/no) | Binary | 0=No  1= Yes |
| PAX1 methylation | Binary | 0=>6  1= ≤6 |
| TCT | Categorical | 0=NILM／inflammation  1=≤LSIL  2=>HSIL |
| Cervix visibility | Binary | 0=Adequate  1=Inadequate |
| Cervical atrophy（yes/no） | Binary | 0=No  1= Yes |
| TZ type | Categorical | 1=Type I  2=Type II  3=Type III |
| Acetowhite changes | Categorical | 0=None  1=Thin  2=Dense |
| Lugol staining | Binary | 0=Stained  1=Nonstained |
| Colposcopic impression  (higest grade) | Categorical | 0=Normal/benign  1=Low-grade  2=High-grade  3=Cancer |
| Contraception | Categorical | 0=None  1=Condom  2=Contraceptive rings  3=Ligation  4=Drug |
